# Bis(μ_2_-2-chloro­benzoato-κ^2^ *O*:*O*′)bis­[(2-chloro­benzoato-κ*O*)(1,10-phenanthroline-κ^2^ *N*:*N*′)copper(II)] dihydrate

**DOI:** 10.1107/S1600536811029709

**Published:** 2011-07-30

**Authors:** Zhi-Fang Zhang

**Affiliations:** a School of Chemistry and Chemical Engineering, Yulin University, Yulin 719000, People’s Republic of China

## Abstract

In the title compound, [Cu_2_(C_7_H_4_ClO_2_)_4_(C_12_H_8_N_2_)_2_]·2H_2_O, the two crystallographically independent dinuclear complex mol­ecules, *A* and *B*, have different Cu⋯Cu separations, *viz.* 3.286 (1) Å in *A* and 3.451 (1) Å in *B*. Both independent mol­ecules reside on inversion centres, so the asymmetric unit contains a half-mol­ecule each of *A* and *B* and two water mol­ecules. Each Cu atom has a square-pyramidal environment, being coordinated by two O atoms from two bridging 2-chloro­benzoate ligands, one O atom from a monodentate 2-chloro­benzoate ligand and two N atoms from a 1,10-phenanthroline ligand. The water mol­ecules can also be considered as coordinating ligands, which complete the coordination geometry up to distorted octa­hedral with elongated Cu—O distances, *viz.* 3.024 (3) Å in *A* and 2.917 (3) Å in *B*. In the crystal, weak inter­molecular C—H⋯O inter­actions contribute to the consolidation of the crystal packing.

## Related literature

For applications of copper complexes, see: Lo *et al.* (2000 ▶); Müller *et al.* (2003 ▶); Rao *et al.* (2004 ▶).
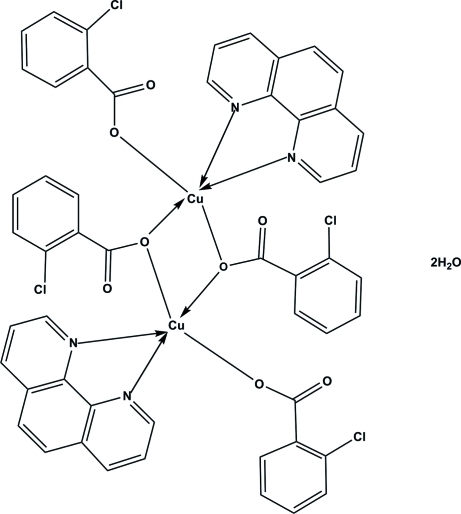


## Experimental

### 

#### Crystal data

[Cu_2_(C_7_H_4_ClO_2_)_4_(C_12_H_8_N_2_)_2_]·2H_2_O
*M* *_r_* = 1145.75
Triclinic, 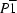

*a* = 10.434 (2) Å
*b* = 11.726 (2) Å
*c* = 22.200 (4) Å
α = 100.90 (3)°
β = 93.92 (2)°
γ = 111.62 (3)°
*V* = 2451.0 (10) Å^3^
*Z* = 2
Mo *K*α radiation
μ = 1.15 mm^−1^
*T* = 295 K
0.13 × 0.10 × 0.08 mm

#### Data collection

Bruker APEXII CCD diffractometer
Absorption correction: multi-scan (*SADABS*; Sheldrick, 2003 ▶) *T*_min_ = 0.865, *T*_max_ = 0.914
12840 measured reflections
8587 independent reflections
6410 reflections with *I* > 2σ(*I*)
*R*_int_ = 0.018

#### Refinement

*R*[*F* ^2^ > 2σ(*F* ^2^)] = 0.041
*wR*(*F* ^2^) = 0.117
*S* = 1.04
8587 reflections
649 parameters
8 restraints
H-atom parameters constrained
Δρ_max_ = 0.41 e Å^−3^
Δρ_min_ = −0.42 e Å^−3^

### 

Data collection: *APEX2* (Bruker, 2007 ▶); cell refinement: *SAINT-Plus* (Bruker, 2007 ▶); data reduction: *SAINT-Plus*; program(s) used to solve structure: *SHELXTL* (Sheldrick, 2008 ▶); program(s) used to refine structure: *SHELXTL*; molecular graphics: *SHELXTL*; software used to prepare material for publication: *SHELXTL*.

## Supplementary Material

Crystal structure: contains datablock(s) I, global. DOI: 10.1107/S1600536811029709/cv5135sup1.cif

Structure factors: contains datablock(s) I. DOI: 10.1107/S1600536811029709/cv5135Isup2.hkl

Additional supplementary materials:  crystallographic information; 3D view; checkCIF report

## Figures and Tables

**Table 1:** Hydrogen-bond geometry (Å, °)

*D*—H⋯*A*	*D*—H	H⋯*A*	*D*⋯*A*	*D*—H⋯*A*
C3—H3⋯O9^i^	0.93	2.53	3.267 (6)	136
C8—H8⋯O2^ii^	0.93	2.47	3.182 (5)	134
C29—H29⋯O6^iii^	0.93	2.52	3.396 (5)	157
C34—H34⋯O8^iv^	0.93	2.52	3.376 (5)	153
C36—H36⋯O5^v^	0.93	2.49	3.054 (4)	119
C42—H42⋯O2^vi^	0.93	2.48	3.338 (5)	153
O9—H9*A*⋯O4	0.85	1.77	2.616 (4)	179
O9—H9*B*⋯O2	0.85	2.04	2.826 (4)	154
O10—H10*A*⋯O6	0.85	2.03	2.852 (5)	162
O10—H10*B*⋯O8	0.88	2.16	2.928 (5)	145

Symmetry codes: (i) 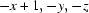; (ii) 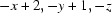; (iii) 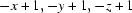; (iv) 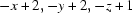; (v) 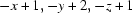; (vi) 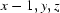.

**Fig. 1.**
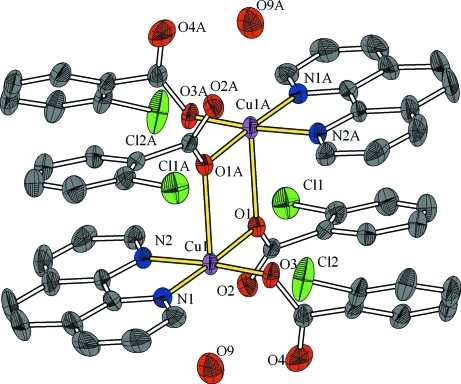
View of the molecule A in (I) showing the atomic numbering of non-C atoms and 30% probability displacement ellipsoids [symmetry code: (A) 1 - x, 1 - y, -z]. H atoms omitted for clarity.

**Fig. 2.**
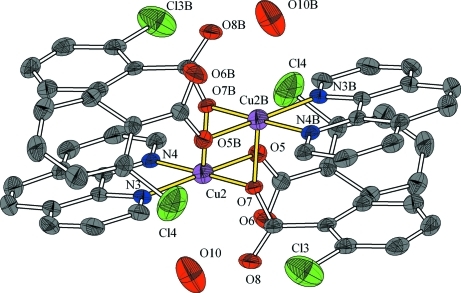
View of the molecule B in (I) showing the atomic numbering of non-C atoms and 30% probability displacement ellipsoids [symmetry code: (B) 1 - x, 2 - y, 1 - z]. H atoms omitted for clarity.

## supplementary crystallographic information

### Comment

During the last three decades, copper complexes have received much attention because of their interesting interactions with biological ligands to generate stable mixed coordinated complexes, which play a key role in life processes such as enzymatic catalysis, storage and conveyance of the matter, transfer of copper ions (Müller *et al.*, 2003; Rao *et al.*, 2004; Lo *et al*., 2000). In order to extend further the study of benzoic acid ligand coordinated to copper ion, we have synthesized the title compound and determined its crystal structure.

The title compound, (I), contains two crystallographically independent dinuclear complex molecules, *A* (Fig. 1) and *B* (Fig. 2), respectively, with different Cu···Cu separation - 3.286 (1) Å in *A* and 3.451 (1) Å in *B*. Both independent molecules reside on inversion centres, so asymmetric part contains a half of the *A* molecule, half of the *B* molecule and two water molecules. Each copper center has a square pyramidal environment being coordinated by two O atoms from two bridging 2-chlorobenzoato ligands, one O atom from monodentate 2-chlorobenzoato ligand and two N atoms from 1,10-phenanthroline ligand. The water molecules can be considered as coordinating ligands too, which complete the coordination geometry up to distorted octahedron with elongated Cu—O distances - 3.024 (3) Å in *A* and 2.917 (3) Å in *B*.

In the crystal structure, weak intermolecular C—H···O interactions (Table 1) contribute to the crystal packing consolidation.

### Experimental

All reagents were obtained from commercial sources and used without further purification. Cu(OAc)_2_.H_2_O (0.1996 g,0.10 mmol) was added to 30 ml CH_3_OH—H_2_O (2:1,*v*/*v*) solution. Then 2-chlorobenzoic acid (0.3131 g, 0.20 mmol) and 1,10-phenanthroline(0.1980 g, 0.10 mmol) were subsequently added. The pH value of the mixture was adjusted to about 5.5 with NaOH solution and stirred continuously for 2 h to obtain a blue clear solution. After filtration, the blue filtrate was allowed to stand at room temperature for two weeks to produce blue block-shaped crystals suitable for X-ray analysis.

### Refinement

H atoms were positioned geometrically (O—H 0.85 Å; C—H 0.93 Å), and refined as riding, with *U*iso(H) = 1.2-1.5 *U*eq (C).

### Crystal data

**Table:**

[Cu_2_(C_7_H_4_ClO_2_)_4_(C_12_H_8_N_2_)_2_]·2H_2_O	*Z* = 2
*M**_r_* = 1145.75	*F*(000) = 582
Triclinic, *P*1	*D*_x_ = 1.553 Mg m^−^^3^
Hall symbol: -P 1	Mo *K*α radiation, λ = 0.71073 Å
*a* = 10.434 (2) Å	Cell parameters from 3998 reflections
*b* = 11.726 (2) Å	θ = 2.2–25.4°
*c* = 22.200 (4) Å	µ = 1.15 mm^−^^1^
α = 100.90 (3)°	*T* = 295 K
β = 93.92 (2)°	Block, blue
γ = 111.62 (3)°	0.13 × 0.10 × 0.08 mm
*V* = 2451.0 (10) Å^3^	

### Data collection

**Table:**

Bruker APEXII CCD diffractometer	8587 independent reflections
Radiation source: fine-focus sealed tube	6410 reflections with *I* > 2σ(*I*)
graphite	*R*_int_ = 0.018
φ and ω scans	θ_max_ = 25.1°, θ_min_ = 0.9°
Absorption correction: multi-scan (*SADABS*; Sheldrick, 2003)	*h* = −12→12
*T*_min_ = 0.865, *T*_max_ = 0.914	*k* = −13→13
12840 measured reflections	*l* = −26→23

### Refinement

**Table:**

Refinement on *F*^2^	Primary atom site location: structure-invariant direct methods
Least-squares matrix: full	Secondary atom site location: difference Fourier map
*R*[*F*^2^ > 2σ(*F*^2^)] = 0.041	Hydrogen site location: inferred from neighbouring sites
*wR*(*F*^2^) = 0.117	H-atom parameters constrained
*S* = 1.04	*w* = 1/[σ^2^(*F*_o_^2^) + (0.0661*P*)^2^ + 0.0501*P*] where *P* = (*F*_o_^2^ + 2*F*_c_^2^)/3
8587 reflections	(Δ/σ)_max_ = 0.001
649 parameters	Δρ_max_ = 0.41 e Å^−^^3^
8 restraints	Δρ_min_ = −0.42 e Å^−^^3^

### Special details

**Table:**

Geometry. All e.s.d.'s (except the e.s.d. in the dihedral angle between two l.s. planes) are estimated using the full covariance matrix. The cell e.s.d.'s are taken into account individually in the estimation of e.s.d.'s in distances, angles and torsion angles; correlations between e.s.d.'s in cell parameters are only used when they are defined by crystal symmetry. An approximate (isotropic) treatment of cell e.s.d.'s is used for estimating e.s.d.'s involving l.s. planes.
Refinement. Refinement of *F*^2^ against ALL reflections. The weighted *R*-factor *wR* and goodness of fit *S* are based on *F*^2^, conventional *R*-factors *R* are based on *F*, with *F* set to zero for negative *F*^2^. The threshold expression of *F*^2^ > σ(*F*^2^) is used only for calculating *R*-factors(gt) *etc*. and is not relevant to the choice of reflections for refinement. *R*-factors based on *F*^2^ are statistically about twice as large as those based on *F*, and *R*- factors based on ALL data will be even larger.

### Fractional atomic coordinates and isotropic or equivalent isotropic displacement parameters (Å^2^)

**Table:**

	*x*	*y*	*z*	*U*_iso_*/*U*_eq_	
Cu1	0.54184 (4)	0.38049 (4)	0.018837 (18)	0.03958 (13)	
Cl1	0.95390 (11)	0.90781 (10)	0.12910 (6)	0.0788 (3)	
Cl2	0.09359 (13)	0.15601 (12)	0.05237 (6)	0.0980 (5)	
O1	0.6047 (2)	0.56208 (19)	0.05751 (10)	0.0376 (5)	
O2	0.8306 (2)	0.6137 (2)	0.11067 (11)	0.0521 (6)	
O3	0.3988 (2)	0.3439 (2)	0.06436 (11)	0.0504 (6)	
O4	0.5292 (3)	0.3361 (4)	0.15246 (15)	0.0926 (11)	
O9	0.7523 (3)	0.3525 (3)	0.10158 (15)	0.0870 (10)	
H9A	0.6802	0.3471	0.1184	0.130*	
H9B	0.8018	0.4299	0.1045	0.130*	
N1	0.4697 (3)	0.1959 (3)	−0.02423 (13)	0.0442 (7)	
N2	0.6837 (3)	0.3972 (3)	−0.03053 (13)	0.0435 (7)	
C1	0.3616 (4)	0.0977 (3)	−0.01969 (18)	0.0542 (10)	
H1	0.3026	0.1123	0.0076	0.065*	
C2	0.3260 (5)	−0.0269 (4)	−0.0519 (2)	0.0717 (13)	
H2	0.2478	−0.0916	−0.0454	0.086*	
C3	0.4021 (6)	−0.0494 (4)	−0.0899 (2)	0.0816 (15)	
H3	0.3815	−0.1305	−0.1130	0.098*	
C4	0.5178 (5)	0.0518 (5)	−0.09597 (19)	0.0703 (13)	
C5	0.6086 (7)	0.0408 (6)	−0.1339 (3)	0.1012 (19)	
H5	0.5947	−0.0377	−0.1583	0.121*	
C6	0.7181 (7)	0.1411 (7)	−0.1370 (3)	0.104 (2)	
H6	0.7794	0.1290	−0.1636	0.125*	
C7	0.7503 (5)	0.2677 (5)	−0.1023 (2)	0.0693 (12)	
C8	0.8621 (5)	0.3766 (6)	−0.1033 (2)	0.0865 (16)	
H8	0.9268	0.3699	−0.1292	0.104*	
C9	0.8838 (4)	0.4928 (5)	−0.0691 (2)	0.0746 (14)	
H9	0.9608	0.5637	−0.0709	0.090*	
C10	0.7901 (4)	0.5001 (4)	−0.03298 (18)	0.0550 (10)	
H10	0.7992	0.5779	−0.0093	0.066*	
C11	0.6638 (4)	0.2825 (4)	−0.06467 (16)	0.0495 (9)	
C12	0.5485 (4)	0.1746 (4)	−0.06165 (17)	0.0495 (9)	
C13	0.4217 (4)	0.3373 (3)	0.12021 (18)	0.0489 (9)	
C14	0.3049 (4)	0.3387 (3)	0.14993 (15)	0.0445 (8)	
C15	0.1598 (4)	0.2647 (4)	0.12444 (17)	0.0575 (10)	
C16	0.0608 (4)	0.2747 (4)	0.1542 (2)	0.0713 (12)	
H16	−0.0330	0.2265	0.1392	0.086*	
C17	0.1057 (5)	0.3630 (4)	0.2102 (2)	0.0747 (13)	
H17	0.0378	0.3751	0.2326	0.090*	
C18	0.2490 (5)	0.4375 (4)	0.23637 (19)	0.0680 (12)	
H18	0.2708	0.4955	0.2742	0.082*	
C19	0.3469 (4)	0.4241 (4)	0.20759 (17)	0.0551 (10)	
H19	0.4405	0.4687	0.2242	0.066*	
C20	0.7204 (3)	0.6319 (3)	0.10087 (14)	0.0368 (7)	
C21	0.7129 (3)	0.7380 (3)	0.14494 (15)	0.0389 (8)	
C22	0.6016 (4)	0.7086 (4)	0.17226 (17)	0.0529 (9)	
H22	0.5299	0.6291	0.1603	0.063*	
C23	0.5954 (5)	0.7976 (5)	0.2179 (2)	0.0740 (13)	
H23	0.5183	0.7788	0.2386	0.089*	
C24	0.7025 (6)	0.9198 (5)	0.2359 (2)	0.0844 (15)	
H24	0.6942	0.9782	0.2684	0.101*	
C25	0.8105 (5)	0.9526 (4)	0.2088 (2)	0.0703 (12)	
H25	0.8796	1.0335	0.2196	0.084*	
C26	0.8166 (4)	0.8620 (3)	0.16380 (17)	0.0497 (9)	
Cu2	0.52065 (4)	0.86567 (4)	0.485627 (18)	0.03791 (13)	
Cl3	0.73450 (12)	1.24915 (11)	0.35243 (7)	0.0906 (4)	
Cl4	0.02279 (12)	0.59677 (15)	0.45077 (6)	0.0978 (5)	
O5	0.3161 (2)	0.7761 (2)	0.44333 (11)	0.0488 (6)	
O6	0.3543 (3)	0.6717 (3)	0.36542 (14)	0.0828 (10)	
O7	0.5251 (2)	0.9980 (2)	0.44242 (10)	0.0411 (5)	
O8	0.6558 (3)	0.9789 (3)	0.37891 (13)	0.0726 (9)	
N3	0.7321 (3)	0.9371 (3)	0.52815 (13)	0.0425 (7)	
N4	0.5171 (3)	0.7286 (3)	0.52843 (12)	0.0417 (7)	
C27	0.4072 (4)	0.6285 (3)	0.52898 (16)	0.0494 (9)	
H27	0.3204	0.6164	0.5089	0.059*	
C28	0.4216 (5)	0.5387 (4)	0.56053 (18)	0.0613 (11)	
H28	0.3434	0.4676	0.5612	0.074*	
C29	0.5561 (5)	0.5555 (4)	0.59189 (19)	0.0664 (12)	
H29	0.5628	0.4948	0.6121	0.080*	
C30	0.6754 (5)	0.6610 (4)	0.59229 (17)	0.0578 (10)	
C31	0.8209 (5)	0.6897 (5)	0.6241 (2)	0.0757 (13)	
H31	0.8350	0.6315	0.6441	0.091*	
C32	0.9289 (5)	0.7951 (6)	0.6245 (2)	0.0789 (14)	
H32	1.0171	0.8097	0.6439	0.095*	
C33	0.9069 (4)	0.8875 (4)	0.59384 (18)	0.0563 (10)	
C34	1.0139 (4)	1.0030 (5)	0.5946 (2)	0.0681 (12)	
H34	1.1038	1.0258	0.6149	0.082*	
C35	0.9785 (4)	1.0814 (4)	0.5635 (2)	0.0650 (11)	
H35	1.0466	1.1595	0.5633	0.078*	
C36	0.8359 (4)	1.0457 (4)	0.53021 (18)	0.0518 (9)	
H36	0.8187	1.1023	0.5099	0.062*	
C37	0.7663 (4)	0.8591 (3)	0.56016 (15)	0.0447 (8)	
C38	0.6505 (4)	0.7466 (3)	0.55994 (15)	0.0437 (8)	
C39	0.2722 (3)	0.7018 (3)	0.38904 (16)	0.0449 (8)	
C40	0.1253 (3)	0.6602 (3)	0.35420 (15)	0.0440 (8)	
C41	0.0958 (4)	0.6676 (4)	0.29241 (17)	0.0605 (10)	
H41	0.1724	0.6946	0.2719	0.073*	
C42	−0.0403 (5)	0.6381 (5)	0.2565 (2)	0.0825 (14)	
H42	−0.0448	0.6506	0.2164	0.099*	
C43	−0.1542 (5)	0.5948 (5)	0.2797 (2)	0.0855 (15)	
H43	−0.2421	0.5750	0.2581	0.103*	
C44	−0.1337 (4)	0.5809 (5)	0.3391 (2)	0.0795 (14)	
H44	−0.2122	0.5477	0.3577	0.095*	
C45	0.0045 (4)	0.6149 (4)	0.37574 (18)	0.0573 (10)	
C46	0.5634 (3)	1.0106 (3)	0.39248 (14)	0.0438 (8)	
C47	0.4857 (3)	1.0618 (3)	0.35128 (14)	0.0427 (8)	
C48	0.3355 (4)	1.0019 (3)	0.33068 (16)	0.0476 (9)	
H48	0.2861	0.9328	0.3463	0.057*	
C49	0.2565 (5)	1.0395 (4)	0.28870 (18)	0.0604 (10)	
H49	0.1607	0.9956	0.2776	0.072*	
C50	0.3245 (5)	1.1393 (4)	0.26607 (19)	0.0695 (12)	
H50	0.2773	1.1665	0.2386	0.083*	
C51	0.4700 (5)	1.2025 (4)	0.28511 (19)	0.0650 (12)	
H51	0.5173	1.2727	0.2699	0.078*	
C52	0.5492 (4)	1.1635 (3)	0.32706 (18)	0.0542 (10)	
O10	0.6462 (4)	0.7321 (4)	0.3920 (2)	0.1380 (18)	
H10A	0.5620	0.7100	0.3758	0.207*	
H10B	0.6858	0.8140	0.3956	0.207*	

### Atomic displacement parameters (Å^2^)

**Table:**

	*U*^11^	*U*^22^	*U*^33^	*U*^12^	*U*^13^	*U*^23^
Cu1	0.0460 (2)	0.0370 (2)	0.0378 (2)	0.01726 (19)	0.01944 (19)	0.00650 (18)
Cl1	0.0632 (7)	0.0623 (7)	0.0957 (9)	0.0055 (5)	0.0268 (6)	0.0168 (6)
Cl2	0.0783 (8)	0.0894 (9)	0.0672 (8)	−0.0183 (7)	0.0358 (6)	−0.0230 (6)
O1	0.0396 (12)	0.0353 (12)	0.0355 (12)	0.0147 (10)	0.0072 (10)	0.0018 (10)
O2	0.0480 (14)	0.0628 (16)	0.0484 (15)	0.0295 (13)	0.0057 (12)	0.0037 (12)
O3	0.0544 (15)	0.0547 (15)	0.0418 (15)	0.0181 (12)	0.0259 (12)	0.0104 (12)
O4	0.082 (2)	0.157 (3)	0.078 (2)	0.071 (2)	0.0393 (19)	0.060 (2)
O9	0.099 (2)	0.067 (2)	0.098 (3)	0.0319 (18)	0.037 (2)	0.0200 (18)
N1	0.0589 (18)	0.0427 (17)	0.0379 (16)	0.0266 (15)	0.0120 (15)	0.0097 (13)
N2	0.0445 (16)	0.0560 (19)	0.0392 (16)	0.0271 (15)	0.0162 (14)	0.0137 (14)
C1	0.067 (3)	0.043 (2)	0.050 (2)	0.021 (2)	0.003 (2)	0.0103 (18)
C2	0.093 (3)	0.043 (2)	0.069 (3)	0.022 (2)	−0.010 (3)	0.005 (2)
C3	0.123 (4)	0.050 (3)	0.072 (3)	0.048 (3)	−0.009 (3)	−0.008 (2)
C4	0.106 (4)	0.072 (3)	0.049 (2)	0.062 (3)	0.004 (3)	−0.004 (2)
C5	0.148 (6)	0.110 (5)	0.077 (4)	0.096 (5)	0.029 (4)	−0.006 (3)
C6	0.125 (5)	0.144 (6)	0.080 (4)	0.102 (5)	0.041 (4)	0.000 (4)
C7	0.066 (3)	0.109 (4)	0.052 (3)	0.057 (3)	0.022 (2)	0.011 (3)
C8	0.075 (3)	0.152 (5)	0.059 (3)	0.072 (4)	0.032 (3)	0.018 (3)
C9	0.051 (2)	0.124 (4)	0.067 (3)	0.039 (3)	0.029 (2)	0.046 (3)
C10	0.049 (2)	0.076 (3)	0.051 (2)	0.030 (2)	0.0213 (19)	0.023 (2)
C11	0.059 (2)	0.073 (3)	0.036 (2)	0.047 (2)	0.0160 (18)	0.0115 (19)
C12	0.065 (2)	0.057 (2)	0.041 (2)	0.042 (2)	0.0079 (19)	0.0061 (18)
C13	0.060 (2)	0.039 (2)	0.049 (2)	0.0171 (18)	0.0242 (19)	0.0140 (17)
C14	0.059 (2)	0.0406 (19)	0.0339 (19)	0.0157 (17)	0.0184 (17)	0.0129 (16)
C15	0.066 (3)	0.052 (2)	0.043 (2)	0.009 (2)	0.026 (2)	0.0070 (18)
C16	0.058 (3)	0.084 (3)	0.059 (3)	0.010 (2)	0.033 (2)	0.015 (2)
C17	0.087 (3)	0.086 (3)	0.059 (3)	0.035 (3)	0.045 (3)	0.020 (3)
C18	0.098 (3)	0.066 (3)	0.042 (2)	0.033 (3)	0.031 (2)	0.008 (2)
C19	0.066 (2)	0.055 (2)	0.044 (2)	0.019 (2)	0.018 (2)	0.0143 (18)
C20	0.0441 (19)	0.0379 (18)	0.0302 (18)	0.0161 (16)	0.0122 (15)	0.0096 (15)
C21	0.0467 (19)	0.044 (2)	0.0282 (17)	0.0209 (16)	0.0053 (15)	0.0078 (15)
C22	0.066 (2)	0.058 (2)	0.048 (2)	0.034 (2)	0.024 (2)	0.0167 (19)
C23	0.100 (4)	0.092 (4)	0.054 (3)	0.058 (3)	0.038 (3)	0.022 (3)
C24	0.134 (5)	0.088 (4)	0.048 (3)	0.069 (4)	0.022 (3)	0.000 (3)
C25	0.092 (3)	0.051 (3)	0.056 (3)	0.025 (2)	0.003 (3)	−0.007 (2)
C26	0.054 (2)	0.048 (2)	0.041 (2)	0.0167 (18)	0.0020 (18)	0.0033 (17)
Cu2	0.0422 (2)	0.0414 (2)	0.0336 (2)	0.0207 (2)	0.00609 (18)	0.00762 (18)
Cl3	0.0742 (8)	0.0713 (8)	0.1372 (12)	0.0275 (6)	0.0475 (8)	0.0399 (8)
Cl4	0.0716 (8)	0.1367 (12)	0.0786 (9)	0.0123 (8)	0.0162 (7)	0.0663 (9)
O5	0.0462 (14)	0.0533 (15)	0.0423 (14)	0.0178 (12)	0.0024 (12)	0.0049 (12)
O6	0.0676 (19)	0.105 (3)	0.074 (2)	0.0526 (19)	0.0047 (17)	−0.0177 (18)
O7	0.0555 (14)	0.0489 (14)	0.0328 (13)	0.0314 (12)	0.0185 (11)	0.0146 (10)
O8	0.093 (2)	0.104 (2)	0.0677 (19)	0.074 (2)	0.0489 (17)	0.0417 (17)
N3	0.0436 (16)	0.0514 (18)	0.0385 (16)	0.0264 (15)	0.0128 (13)	0.0059 (14)
N4	0.0538 (17)	0.0443 (17)	0.0321 (15)	0.0259 (15)	0.0128 (13)	0.0042 (13)
C27	0.064 (2)	0.048 (2)	0.040 (2)	0.027 (2)	0.0144 (18)	0.0071 (17)
C28	0.088 (3)	0.051 (2)	0.052 (2)	0.030 (2)	0.029 (2)	0.0144 (19)
C29	0.106 (4)	0.065 (3)	0.054 (3)	0.054 (3)	0.030 (3)	0.025 (2)
C30	0.087 (3)	0.069 (3)	0.039 (2)	0.053 (3)	0.017 (2)	0.014 (2)
C31	0.098 (4)	0.098 (4)	0.066 (3)	0.069 (3)	0.020 (3)	0.034 (3)
C32	0.080 (3)	0.129 (5)	0.062 (3)	0.075 (3)	0.014 (3)	0.029 (3)
C33	0.057 (2)	0.082 (3)	0.043 (2)	0.044 (2)	0.0116 (19)	0.008 (2)
C34	0.042 (2)	0.105 (4)	0.058 (3)	0.036 (3)	0.007 (2)	0.006 (3)
C35	0.049 (2)	0.077 (3)	0.063 (3)	0.023 (2)	0.018 (2)	0.002 (2)
C36	0.047 (2)	0.057 (2)	0.052 (2)	0.0227 (19)	0.0167 (18)	0.0057 (19)
C37	0.050 (2)	0.062 (2)	0.0339 (19)	0.0371 (19)	0.0116 (16)	0.0052 (17)
C38	0.059 (2)	0.051 (2)	0.0322 (18)	0.0353 (19)	0.0110 (17)	0.0051 (16)
C39	0.053 (2)	0.041 (2)	0.044 (2)	0.0232 (18)	0.0069 (18)	0.0070 (17)
C40	0.049 (2)	0.041 (2)	0.041 (2)	0.0180 (17)	0.0018 (17)	0.0064 (16)
C41	0.062 (2)	0.079 (3)	0.039 (2)	0.032 (2)	0.0057 (19)	0.003 (2)
C42	0.076 (3)	0.123 (4)	0.048 (3)	0.042 (3)	−0.002 (2)	0.015 (3)
C43	0.052 (3)	0.121 (4)	0.072 (3)	0.026 (3)	−0.015 (3)	0.020 (3)
C44	0.050 (2)	0.097 (4)	0.084 (4)	0.015 (2)	0.008 (2)	0.031 (3)
C45	0.052 (2)	0.058 (2)	0.054 (2)	0.0102 (19)	0.0026 (19)	0.022 (2)
C46	0.056 (2)	0.046 (2)	0.037 (2)	0.0273 (18)	0.0175 (17)	0.0078 (16)
C47	0.061 (2)	0.047 (2)	0.0304 (18)	0.0310 (18)	0.0188 (17)	0.0082 (15)
C48	0.062 (2)	0.050 (2)	0.040 (2)	0.0312 (19)	0.0172 (18)	0.0091 (17)
C49	0.071 (3)	0.071 (3)	0.046 (2)	0.039 (2)	0.009 (2)	0.006 (2)
C50	0.111 (4)	0.081 (3)	0.047 (2)	0.067 (3)	0.019 (3)	0.023 (2)
C51	0.100 (3)	0.062 (3)	0.059 (3)	0.048 (3)	0.039 (3)	0.031 (2)
C52	0.076 (3)	0.052 (2)	0.051 (2)	0.037 (2)	0.031 (2)	0.0166 (19)
O10	0.081 (2)	0.122 (3)	0.238 (6)	0.037 (2)	0.036 (3)	0.105 (4)

### Geometric parameters (Å, °)

**Table:**

Cu1—O3	1.825 (2)	Cu2—N4	2.006 (3)
Cu1—N2	1.876 (3)	Cu2—O5	2.060 (3)
Cu1—O1	1.976 (2)	Cu2—N3	2.125 (3)
Cu1—N1	2.021 (3)	Cu2—O7^i^	2.244 (2)
Cl1—C26	1.636 (4)	Cl3—C52	1.809 (4)
Cl2—C15	1.758 (4)	Cl4—C45	1.725 (4)
O1—C20	1.367 (4)	O5—C39	1.286 (4)
O2—C20	1.257 (4)	O6—C39	1.162 (4)
O3—C13	1.269 (4)	O7—C46	1.220 (4)
O4—C13	1.295 (5)	O7—Cu2^i^	2.244 (2)
O9—H9A	0.8496	O8—C46	1.193 (4)
O9—H9B	0.8500	N3—C36	1.323 (4)
N1—C12	1.262 (4)	N3—C37	1.386 (4)
N1—C1	1.308 (4)	N4—C27	1.308 (4)
N2—C10	1.318 (5)	N4—C38	1.438 (4)
N2—C11	1.346 (5)	C27—C28	1.415 (5)
C1—C2	1.398 (5)	C27—H27	0.9300
C1—H1	0.9300	C28—C29	1.450 (6)
C2—C3	1.253 (6)	C28—H28	0.9300
C2—H2	0.9300	C29—C30	1.393 (6)
C3—C4	1.382 (7)	C29—H29	0.9300
C3—H3	0.9300	C30—C38	1.424 (5)
C4—C5	1.335 (7)	C30—C31	1.518 (6)
C4—C12	1.403 (5)	C31—C32	1.328 (6)
C5—C6	1.320 (8)	C31—H31	0.9300
C5—H5	0.9300	C32—C33	1.460 (6)
C6—C7	1.440 (7)	C32—H32	0.9300
C6—H6	0.9300	C33—C34	1.399 (6)
C7—C11	1.307 (5)	C33—C37	1.489 (5)
C7—C8	1.382 (7)	C34—C35	1.385 (6)
C8—C9	1.357 (7)	C34—H34	0.9300
C8—H8	0.9300	C35—C36	1.486 (5)
C9—C10	1.322 (5)	C35—H35	0.9300
C9—H9	0.9300	C36—H36	0.9300
C10—H10	0.9300	C37—C38	1.422 (5)
C11—C12	1.406 (5)	C39—C40	1.526 (2)
C13—C14	1.429 (5)	C40—C45	1.337 (5)
C14—C19	1.396 (5)	C40—C41	1.411 (5)
C14—C15	1.448 (5)	C41—C42	1.467 (6)
C15—C16	1.293 (5)	C41—H41	0.9300
C16—C17	1.382 (6)	C42—C43	1.294 (6)
C16—H16	0.9300	C42—H42	0.9300
C17—C18	1.436 (6)	C43—C44	1.371 (6)
C17—H17	0.9300	C43—H43	0.9300
C18—C19	1.283 (5)	C44—C45	1.481 (6)
C18—H18	0.9300	C44—H44	0.9300
C19—H19	0.9300	C46—C47	1.522 (2)
C20—C21	1.460 (4)	C47—C52	1.364 (5)
C21—C22	1.310 (5)	C47—C48	1.459 (5)
C21—C26	1.417 (5)	C48—C49	1.435 (5)
C22—C23	1.334 (5)	C48—H48	0.9300
C22—H22	0.9300	C49—C50	1.336 (6)
C23—C24	1.415 (7)	C49—H49	0.9300
C23—H23	0.9300	C50—C51	1.414 (6)
C24—C25	1.280 (6)	C50—H50	0.9300
C24—H24	0.9300	C51—C52	1.447 (6)
C25—C26	1.337 (5)	C51—H51	0.9300
C25—H25	0.9300	O10—H10A	0.8501
Cu2—O7	1.961 (2)	O10—H10B	0.8796
			
O3—Cu1—N2	173.10 (12)	O7—Cu2—N3	100.53 (11)
O3—Cu1—O1	90.74 (10)	N4—Cu2—N3	79.43 (12)
N2—Cu1—O1	96.02 (11)	O5—Cu2—N3	173.41 (10)
O3—Cu1—N1	88.95 (11)	O7—Cu2—O7^i^	77.45 (9)
N2—Cu1—N1	84.37 (12)	N4—Cu2—O7^i^	103.63 (9)
O1—Cu1—N1	176.75 (10)	O5—Cu2—O7^i^	93.53 (10)
C20—O1—Cu1	125.85 (19)	N3—Cu2—O7^i^	91.28 (10)
C13—O3—Cu1	119.8 (2)	C39—O5—Cu2	126.2 (2)
H9A—O9—H9B	108.7	C46—O7—Cu2	127.7 (2)
C12—N1—C1	116.1 (3)	C46—O7—Cu2^i^	129.0 (2)
C12—N1—Cu1	111.3 (3)	Cu2—O7—Cu2^i^	102.55 (9)
C1—N1—Cu1	132.6 (3)	C36—N3—C37	114.7 (3)
C10—N2—C11	122.5 (3)	C36—N3—Cu2	130.3 (2)
C10—N2—Cu1	128.6 (3)	C37—N3—Cu2	115.0 (2)
C11—N2—Cu1	108.9 (2)	C27—N4—C38	119.8 (3)
N1—C1—C2	126.3 (4)	C27—N4—Cu2	125.8 (2)
N1—C1—H1	116.8	C38—N4—Cu2	114.3 (2)
C2—C1—H1	116.8	N4—C27—C28	119.3 (4)
C3—C2—C1	118.4 (5)	N4—C27—H27	120.4
C3—C2—H2	120.8	C28—C27—H27	120.4
C1—C2—H2	120.8	C27—C28—C29	121.2 (4)
C2—C3—C4	117.1 (4)	C27—C28—H28	119.4
C2—C3—H3	121.4	C29—C28—H28	119.4
C4—C3—H3	121.4	C30—C29—C28	121.1 (3)
C5—C4—C3	123.3 (5)	C30—C29—H29	119.5
C5—C4—C12	115.1 (5)	C28—C29—H29	119.5
C3—C4—C12	121.6 (4)	C29—C30—C38	113.7 (4)
C6—C5—C4	120.2 (5)	C29—C30—C31	125.9 (4)
C6—C5—H5	119.9	C38—C30—C31	120.4 (4)
C4—C5—H5	119.9	C32—C31—C30	122.4 (4)
C5—C6—C7	125.3 (5)	C32—C31—H31	118.8
C5—C6—H6	117.4	C30—C31—H31	118.8
C7—C6—H6	117.4	C31—C32—C33	119.0 (4)
C11—C7—C8	115.2 (4)	C31—C32—H32	120.5
C11—C7—C6	116.4 (5)	C33—C32—H32	120.5
C8—C7—C6	128.4 (4)	C34—C33—C32	122.3 (4)
C9—C8—C7	124.3 (4)	C34—C33—C37	118.1 (3)
C9—C8—H8	117.9	C32—C33—C37	119.6 (4)
C7—C8—H8	117.9	C35—C34—C33	116.2 (4)
C10—C9—C8	116.8 (5)	C35—C34—H34	121.9
C10—C9—H9	121.6	C33—C34—H34	121.9
C8—C9—H9	121.6	C34—C35—C36	122.6 (4)
N2—C10—C9	120.0 (4)	C34—C35—H35	118.7
N2—C10—H10	120.0	C36—C35—H35	118.7
C9—C10—H10	120.0	N3—C36—C35	123.0 (4)
C7—C11—N2	121.2 (4)	N3—C36—H36	118.5
C7—C11—C12	117.5 (4)	C35—C36—H36	118.5
N2—C11—C12	121.3 (3)	N3—C37—C38	113.1 (3)
N1—C12—C4	120.5 (4)	N3—C37—C33	125.4 (3)
N1—C12—C11	114.1 (3)	C38—C37—C33	121.4 (3)
C4—C12—C11	125.4 (4)	C37—C38—C30	117.2 (3)
O3—C13—O4	131.9 (3)	C37—C38—N4	118.0 (3)
O3—C13—C14	108.9 (3)	C30—C38—N4	124.8 (3)
O4—C13—C14	119.1 (3)	O6—C39—O5	115.8 (3)
C19—C14—C13	111.6 (3)	O6—C39—C40	120.2 (3)
C19—C14—C15	122.7 (3)	O5—C39—C40	124.0 (3)
C13—C14—C15	125.7 (3)	C45—C40—C41	108.2 (3)
C16—C15—C14	121.3 (4)	C45—C40—C39	127.6 (3)
C16—C15—Cl2	111.6 (3)	C41—C40—C39	124.2 (3)
C14—C15—Cl2	127.1 (3)	C40—C41—C42	128.3 (4)
C15—C16—C17	114.6 (4)	C40—C41—H41	115.9
C15—C16—H16	122.7	C42—C41—H41	115.9
C17—C16—H16	122.7	C43—C42—C41	121.0 (4)
C16—C17—C18	125.1 (4)	C43—C42—H42	119.5
C16—C17—H17	117.5	C41—C42—H42	119.5
C18—C17—H17	117.5	C42—C43—C44	113.9 (4)
C19—C18—C17	120.1 (4)	C42—C43—H43	123.1
C19—C18—H18	119.9	C44—C43—H43	123.1
C17—C18—H18	119.9	C43—C44—C45	124.7 (4)
C18—C19—C14	116.2 (4)	C43—C44—H44	117.7
C18—C19—H19	121.9	C45—C44—H44	117.7
C14—C19—H19	121.9	C40—C45—C44	123.9 (4)
O2—C20—O1	129.0 (3)	C40—C45—Cl4	113.8 (3)
O2—C20—C21	114.6 (3)	C44—C45—Cl4	122.2 (3)
O1—C20—C21	116.2 (3)	O8—C46—O7	117.8 (3)
C22—C21—C26	118.8 (3)	O8—C46—C47	125.0 (3)
C22—C21—C20	114.1 (3)	O7—C46—C47	117.2 (3)
C26—C21—C20	126.9 (3)	C52—C47—C48	112.9 (3)
C21—C22—C23	117.2 (4)	C52—C47—C46	123.9 (3)
C21—C22—H22	121.4	C48—C47—C46	123.1 (3)
C23—C22—H22	121.4	C49—C48—C47	126.3 (3)
C22—C23—C24	122.0 (4)	C49—C48—H48	116.9
C22—C23—H23	119.0	C47—C48—H48	116.9
C24—C23—H23	119.0	C50—C49—C48	118.2 (4)
C25—C24—C23	122.2 (4)	C50—C49—H49	120.9
C25—C24—H24	118.9	C48—C49—H49	120.9
C23—C24—H24	118.9	C49—C50—C51	118.2 (4)
C24—C25—C26	115.1 (4)	C49—C50—H50	120.9
C24—C25—H25	122.4	C51—C50—H50	120.9
C26—C25—H25	122.4	C50—C51—C52	123.4 (4)
C25—C26—C21	124.5 (4)	C50—C51—H51	118.3
C25—C26—Cl1	113.9 (3)	C52—C51—H51	118.3
C21—C26—Cl1	121.5 (3)	C47—C52—C51	121.1 (4)
O7—Cu2—N4	178.92 (10)	C47—C52—Cl3	116.5 (3)
O7—Cu2—O5	84.93 (10)	C51—C52—Cl3	122.3 (3)
N4—Cu2—O5	95.05 (11)	H10A—O10—H10B	106.0
			
O3—Cu1—O1—C20	106.8 (2)	O5—Cu2—O7—Cu2^i^	94.76 (11)
N2—Cu1—O1—C20	−71.9 (2)	N3—Cu2—O7—Cu2^i^	−88.96 (11)
O1—Cu1—O3—C13	−80.8 (3)	O7^i^—Cu2—O7—Cu2^i^	0.0
N1—Cu1—O3—C13	102.4 (3)	O7—Cu2—N3—C36	5.6 (3)
O3—Cu1—N1—C12	−176.8 (2)	N4—Cu2—N3—C36	−175.5 (3)
N2—Cu1—N1—C12	1.4 (2)	O7^i^—Cu2—N3—C36	−71.8 (3)
O3—Cu1—N1—C1	1.8 (3)	O7—Cu2—N3—C37	−176.2 (2)
N2—Cu1—N1—C1	−179.9 (3)	N4—Cu2—N3—C37	2.7 (2)
O1—Cu1—N2—C10	2.9 (3)	O7^i^—Cu2—N3—C37	106.4 (2)
N1—Cu1—N2—C10	179.6 (3)	O5—Cu2—N4—C27	−5.9 (3)
O1—Cu1—N2—C11	−178.5 (2)	N3—Cu2—N4—C27	177.8 (3)
N1—Cu1—N2—C11	−1.7 (2)	O7^i^—Cu2—N4—C27	89.0 (3)
C12—N1—C1—C2	0.2 (6)	O5—Cu2—N4—C38	173.4 (2)
Cu1—N1—C1—C2	−178.4 (3)	N3—Cu2—N4—C38	−3.0 (2)
N1—C1—C2—C3	−1.2 (7)	O7^i^—Cu2—N4—C38	−91.7 (2)
C1—C2—C3—C4	1.2 (7)	C38—N4—C27—C28	−0.3 (5)
C2—C3—C4—C5	178.9 (5)	Cu2—N4—C27—C28	179.0 (2)
C2—C3—C4—C12	−0.6 (7)	N4—C27—C28—C29	−0.3 (5)
C3—C4—C5—C6	−179.1 (5)	C27—C28—C29—C30	0.4 (6)
C12—C4—C5—C6	0.4 (8)	C28—C29—C30—C38	0.2 (5)
C4—C5—C6—C7	−0.9 (10)	C28—C29—C30—C31	179.1 (4)
C5—C6—C7—C11	0.7 (9)	C29—C30—C31—C32	−177.4 (4)
C5—C6—C7—C8	−179.7 (6)	C38—C30—C31—C32	1.5 (6)
C11—C7—C8—C9	−0.8 (7)	C30—C31—C32—C33	0.8 (7)
C6—C7—C8—C9	179.7 (5)	C31—C32—C33—C34	177.0 (4)
C7—C8—C9—C10	−0.4 (7)	C31—C32—C33—C37	−3.2 (6)
C11—N2—C10—C9	−1.4 (5)	C32—C33—C34—C35	179.8 (4)
Cu1—N2—C10—C9	177.1 (3)	C37—C33—C34—C35	0.0 (5)
C8—C9—C10—N2	1.5 (6)	C33—C34—C35—C36	−0.7 (6)
C8—C7—C11—N2	0.9 (6)	C37—N3—C36—C35	0.7 (5)
C6—C7—C11—N2	−179.4 (4)	Cu2—N3—C36—C35	178.9 (2)
C8—C7—C11—C12	−179.7 (4)	C34—C35—C36—N3	0.4 (6)
C6—C7—C11—C12	−0.1 (6)	C36—N3—C37—C38	176.6 (3)
C10—N2—C11—C7	0.1 (5)	Cu2—N3—C37—C38	−1.9 (3)
Cu1—N2—C11—C7	−178.7 (3)	C36—N3—C37—C33	−1.4 (5)
C10—N2—C11—C12	−179.2 (3)	Cu2—N3—C37—C33	−180.0 (3)
Cu1—N2—C11—C12	2.0 (4)	C34—C33—C37—N3	1.2 (5)
C1—N1—C12—C4	0.5 (5)	C32—C33—C37—N3	−178.6 (3)
Cu1—N1—C12—C4	179.4 (3)	C34—C33—C37—C38	−176.7 (3)
C1—N1—C12—C11	−179.6 (3)	C32—C33—C37—C38	3.5 (5)
Cu1—N1—C12—C11	−0.7 (4)	N3—C37—C38—C30	−179.4 (3)
C5—C4—C12—N1	−179.9 (4)	C33—C37—C38—C30	−1.2 (5)
C3—C4—C12—N1	−0.4 (6)	N3—C37—C38—N4	−0.6 (4)
C5—C4—C12—C11	0.2 (6)	C33—C37—C38—N4	177.5 (3)
C3—C4—C12—C11	179.7 (4)	C29—C30—C38—C37	177.8 (3)
C7—C11—C12—N1	179.8 (3)	C31—C30—C38—C37	−1.2 (5)
N2—C11—C12—N1	−0.9 (5)	C29—C30—C38—N4	−0.9 (5)
C7—C11—C12—C4	−0.3 (6)	C31—C30—C38—N4	−179.8 (3)
N2—C11—C12—C4	179.0 (3)	C27—N4—C38—C37	−177.7 (3)
Cu1—O3—C13—O4	−10.3 (6)	Cu2—N4—C38—C37	3.0 (4)
Cu1—O3—C13—C14	165.9 (2)	C27—N4—C38—C30	0.9 (5)
O3—C13—C14—C19	−132.6 (3)	Cu2—N4—C38—C30	−178.4 (3)
O4—C13—C14—C19	44.1 (5)	Cu2—O5—C39—O6	9.8 (5)
O3—C13—C14—C15	46.1 (5)	Cu2—O5—C39—C40	−166.4 (2)
O4—C13—C14—C15	−137.2 (4)	O6—C39—C40—C45	135.5 (4)
C19—C14—C15—C16	0.3 (6)	O5—C39—C40—C45	−48.5 (5)
C13—C14—C15—C16	−178.2 (4)	O6—C39—C40—C41	−46.5 (5)
C19—C14—C15—Cl2	179.1 (3)	O5—C39—C40—C41	129.5 (4)
C13—C14—C15—Cl2	0.6 (6)	C45—C40—C41—C42	2.7 (6)
C14—C15—C16—C17	1.7 (6)	C39—C40—C41—C42	−175.6 (4)
Cl2—C15—C16—C17	−177.3 (3)	C40—C41—C42—C43	−2.8 (8)
C15—C16—C17—C18	−1.8 (7)	C41—C42—C43—C44	0.2 (8)
C16—C17—C18—C19	−0.5 (7)	C42—C43—C44—C45	1.9 (8)
C17—C18—C19—C14	2.5 (6)	C41—C40—C45—C44	−0.5 (6)
C13—C14—C19—C18	176.2 (3)	C39—C40—C45—C44	177.8 (4)
C15—C14—C19—C18	−2.6 (6)	C41—C40—C45—Cl4	178.6 (3)
Cu1—O1—C20—O2	25.5 (4)	C39—C40—C45—Cl4	−3.2 (5)
Cu1—O1—C20—C21	−149.9 (2)	C43—C44—C45—C40	−1.9 (8)
O2—C20—C21—C22	−125.2 (3)	C43—C44—C45—Cl4	179.1 (4)
O1—C20—C21—C22	50.9 (4)	Cu2—O7—C46—O8	−33.3 (5)
O2—C20—C21—C26	49.2 (5)	Cu2^i^—O7—C46—O8	134.9 (3)
O1—C20—C21—C26	−134.6 (3)	Cu2—O7—C46—C47	144.7 (2)
C26—C21—C22—C23	−2.1 (5)	Cu2^i^—O7—C46—C47	−47.2 (4)
C20—C21—C22—C23	172.9 (3)	O8—C46—C47—C52	−55.7 (5)
C21—C22—C23—C24	1.2 (6)	O7—C46—C47—C52	126.5 (4)
C22—C23—C24—C25	1.0 (8)	O8—C46—C47—C48	120.6 (4)
C23—C24—C25—C26	−2.0 (7)	O7—C46—C47—C48	−57.2 (4)
C24—C25—C26—C21	1.1 (6)	C52—C47—C48—C49	1.1 (5)
C24—C25—C26—Cl1	178.7 (4)	C46—C47—C48—C49	−175.6 (3)
C22—C21—C26—C25	1.0 (6)	C47—C48—C49—C50	−0.6 (6)
C20—C21—C26—C25	−173.2 (4)	C48—C49—C50—C51	−0.4 (6)
C22—C21—C26—Cl1	−176.4 (3)	C49—C50—C51—C52	0.9 (6)
C20—C21—C26—Cl1	9.4 (5)	C48—C47—C52—C51	−0.5 (5)
O7—Cu2—O5—C39	87.2 (3)	C46—C47—C52—C51	176.2 (3)
N4—Cu2—O5—C39	−91.7 (3)	C48—C47—C52—Cl3	177.2 (2)
O7^i^—Cu2—O5—C39	164.3 (3)	C46—C47—C52—Cl3	−6.1 (4)
O5—Cu2—O7—C46	−94.7 (3)	C50—C51—C52—C47	−0.4 (6)
N3—Cu2—O7—C46	81.6 (3)	C50—C51—C52—Cl3	−178.1 (3)
O7^i^—Cu2—O7—C46	170.6 (3)		

Symmetry codes: (i) −*x*+1, −*y*+2, −*z*+1.

### Hydrogen-bond geometry (Å, °)

**Table:**

*D*—H···*A*	*D*—H	H···*A*	*D*···*A*	*D*—H···*A*
C3—H3···O9^ii^	0.93	2.53	3.267 (6)	136.
C8—H8···O2^iii^	0.93	2.47	3.182 (5)	134.
C29—H29···O6^iv^	0.93	2.52	3.396 (5)	157.
C34—H34···O8^v^	0.93	2.52	3.376 (5)	153.
C36—H36···O5^i^	0.93	2.49	3.054 (4)	119.
C42—H42···O2^vi^	0.93	2.48	3.338 (5)	153.
O9—H9A···O4	0.85	1.77	2.616 (4)	179.
O9—H9B···O2	0.85	2.04	2.826 (4)	154.
O10—H10A···O6	0.85	2.03	2.852 (5)	162.
O10—H10B···O8	0.88	2.16	2.928 (5)	145.

Symmetry codes: (ii) −*x*+1, −*y*, −*z*; (iii) −*x*+2, −*y*+1, −*z*; (iv) −*x*+1, −*y*+1, −*z*+1; (v) −*x*+2, −*y*+2, −*z*+1; (i) −*x*+1, −*y*+2, −*z*+1; (vi) *x*−1, *y*, *z*.
